# A Comprehensive Inventory of the Ship Traffic Exhaust Emissions in the Baltic Sea from 2006 to 2009

**DOI:** 10.1007/s13280-013-0389-3

**Published:** 2013-03-12

**Authors:** Jukka-Pekka Jalkanen, Lasse Johansson, Jaakko Kukkonen

**Affiliations:** Air Quality Research, Finnish Meteorological Institute, Erik Palmenin aukio 1, P.O. Box 503, 00101 Helsinki, Finland

**Keywords:** Emission, Shipping, Baltic Sea, STEAM, AIS, SECA

## Abstract

This study addresses the exhaust emissions of CO_2_, NO_*x*_, SO_*x*_, CO, and PM_2.5_ originated from Baltic Sea shipping in 2006–2009. Numerical results have been computed using the Ship Traffic Emissions Assessment Model. This model is based on the messages of the automatic identification system (AIS), which enable the positioning of ships with a high spatial resolution. The NO_*x*_ emissions in 2009 were approximately 7 % higher than in 2006, despite the economic recession. However, the SO_*x*_ emissions in 2009 were approximately 14 % lower, when compared to those in 2006, mainly caused by the fuel requirements of the SO_*x*_ emission control area (SECA) which became effective in May 2006, but affected also by changes in ship activity. Results are presented on the differential geographic distribution of shipping emissions before (Jan–April 2006) and after (Jan–April 2009) the SECA regulations. The predicted NO_*x*_ emissions in 2009 substantially exceeded the emissions in 2006 along major ship routes and at numerous harbors, mostly due to the continuous increase in the number of small vessels that use AIS transmitters. Although the SO_*x*_ emissions have been reduced in 2009 in most major ship routes, these have increased in the vicinity of some harbors and on some densely trafficked routes. A seasonal variation of emissions is also presented, as well as the distribution of emissions in terms of vessel flag state, type, and weight.

## Introduction

There have been discussions in the International Maritime Organization (IMO) regarding various options to curb the exhaust emissions of international shipping. These have addressed primarily climate change impacts and the emissions of CO_2_, but also the emissions of other pollutants, such as NO_*x*_, SO_*x*_, and fine particulate matter (PM_2.5_). In the following, we denote PM_2.5_ simply as PM. According to ENTEC (2005), at least the following criteria are available for allocating the costs of reducing the emissions. First, the emissions of ship traffic could be allocated according to the flag state of the ship (“the polluter pays” principle). Second, the emissions could be allocated according to the geographical area, in which the emissions occur, and third, based on the fuel consumption of ships, which would be derived from the bunker fuel prices. The fourth option is an emission trading system (ETS), which relies on emission credits; this system would resemble the existing CO_2_ credit mechanism that is currently applied in Europe, but does not include yet shipping.

Clearly, each of the proposed cost allocation mechanisms has some limitations. Allocating the emissions based on the flag state could lead to a situation, in which the ships could be reflagged to countries with less strict policies. The flag state allocation would probably only function properly, if it would be done simultaneously globally. In case of the emission allocation based on their geographical distribution, the accurate determination of emissions inside each economic zone would be challenging. The third option of introducing an additional environmental tax to the ship fuel prices is probably the simplest way of allocating the burden, but several administrative questions remain to be resolved, such as, e.g., who will administer the emission funds, which would be collected as a part of the fuel price. The ETS option would function properly only, if the emission targets would be sufficiently strict, and potential credit misuse could be prevented.

There is some uncertainty regarding the fuel sulfur content used onboard the vessels. Berg et al. (2012) presented a methodology to measure gas fluxes of SO_2_ and NO_2_ from ships using optical remote sensing, based on Differential Optical Absorption Spectroscopy. The predictions of a ship emission model, STEAM (used also in this study), were compared with the optical measurements, which showed in case of SO_2_ 18 % overprediction and a correlation coefficient squared (*R* ^2^) of 0.6. In total, 160 measurements for 52 ships were made with Differential Optical Absorption Spectrometer (DOAS). However, the relative uncertainty of the measurement setup was estimated to be substantially high, 40 %. They concluded that a combination of the optical method with modeled power consumption could be used to detect the difference between the emissions of ships running at 1 % and at 0.1 % sulfur content fuels, applicable within the IMO-regulated emission control areas (ECA).

Notteboom (2011) and Jalkanen et al. (2012a) analyzed the impact of the International Maritime Organization’s Tier II/III standards—adopted in October 2008—on costs and prices of roll on/roll off (roro) traffic in the ECA’s in North Europe. They demonstrated that the new Annex VI agreement may be costly for the participants in the shipping industry and will result in higher freight rates.

Sulfur aerosols from shipping can also have a cooling impact on climate, in addition to the economic impacts caused by the higher fuel costs and the detrimental human health effects. Eyring et al. (2010) presented an assessment of the contribution of ocean shipping to anthropogenic emissions, air quality, and climate. The cooling due to altered clouds currently outweighs the warming effects due to greenhouse gases from shipping, overall causing a negative present-day radiative forcing. They concluded that after 50 years the net global mean effect of current shipping emissions would be negligible, caused by cancellation of warming by CO_2_ and cooling by sulfate and nitrogen oxides. Coggon et al. (2012) reported properties of marine aerosol and clouds measured in the shipping lanes between Monterey Bay and San Francisco off the coast of Central California. They concluded that the periods of high aerosol loading were primarily linked to increased ship influence, based on the enhancement of vanadium and cloud droplet number concentrations observed concurrently with a decrease in cloud water pH.

Effects to the environment, for example eutrophication, are also a concern in areas like the Baltic Sea. Bartnicki et al. (2011) used the EMEP/MSC-W model to compute atmospheric nitrogen deposition into the Baltic Sea basin from 1995 to 2006. The annual total nitrogen deposition into the Baltic Sea basin has decreased 13 % during this period, which corresponds with the corresponding total nitrogen emission reduction (11 %) in the HELCOM Contracting Parties. Baltic Sea shipping can contribute significantly to the airborne nitrogen load depending on the geographical location and season, but on annual level its contribution is only about 2–3 % of the total estimated nitrogen input to the Baltic Sea (Bartnicki et al. 2011).

The use of automatic identification system (AIS) data as input for emission modeling has several advantages, compared with the previously presented approaches for evaluating shipping emissions (Jalkanen et al. 2009, 2012b). First, it allows emission predictions of single ships with a high spatial and temporal resolution. It is therefore also easier to evaluate model predictions against individual measurement campaigns, including stack measurements and air quality measurements. The use of AIS data also drastically reduces a major previous uncertainty regarding the times that the ships spend at sea; this factor has a significant impact on overall emissions. The emissions inside harbor areas are also included in AIS-based inventories, although these have commonly been previously neglected (Wang et al. 2007; Eyring et al. 2010). Second, it is possible to study the allocation of shipping emissions according to, e.g., ship types or weights, flag states, and vessel routes, and to present high-resolution geographical distribution of emissions. It has not been possible to make such evaluations with any of the previously presented modeling methods for shipping emissions. Third, it is possible to investigate in detail the changes in emissions caused by various emission reduction measures, policies and strategies, either historically or for scenarios for the future. The main limitations of AIS-based evaluation systems for shipping emissions are that the relevant AIS or shipping technical specification-data may not in all cases be readily available, and that the computations are relatively complex.

The objective of this article is to use a previously developed emission modeling system (Jalkanen et al. 2009, 2012b) to present a comprehensive and detailed emissions inventory from Baltic Sea shipping in 2006–2009. We have, in particular, aimed to evaluate a high-resolution geographical distribution of shipping emissions, and emission allocation by flag state, ship type, and weight. We expect that these results could be useful both (i) for the subsequent evaluation of effects on public health, climate, and the environment, caused by exhaust emissions from marine traffic and (ii) for the consideration of emission reductions by the IMO and other organizations.

## Materials and Methods

### The STEAM2 Model

The emissions of the Baltic Sea shipping were evaluated using an emission modeling program called Ship Traffic Emission Assessment Model, version 2 (STEAM2); for a more detailed description of this model, the reader is referred to Jalkanen et al. (2009, 2012b). The model allows for the influences of travel routes and ship speed, engine load, fuel sulfur content, multiengine setups, abatement methods, and waves (Jalkanen et al. 2012b). This modeling approach uses the position reports generated by the AIS; this system is onboard every vessel of over 300 gross tons in the Baltic Sea. The AIS system provides for automatic updates of the positions and instantaneous speeds of ships at intervals of a few seconds. We have collected and archived all of this data for the whole of the Baltic Sea since 2006.

In addition, the model requires as input the detailed technical specifications of all fuel consuming systems onboard and other relevant technical details of the ships for all the ships considered. Such technical specifications were therefore collected and archived for over 45 000 ships from various sources of information; the data from IHS Fairplay (IHS Fairplay 2012) was the most significant source. The STEAM2 model is then used to combine the AIS-based information with the detailed technical knowledge of the ships, and the model then evaluates instantaneous fuel consumption and emissions of key pollutants for the ships in the Baltic Sea. The fuel consumption and emissions are computed separately for all the vessels in the Baltic Sea area; this results in a regional emission inventory of Baltic Sea shipping.

The model has been able to predict aggregate annual fuel consumption of a collection of large marine ships with a mean prediction error of 9 % (Jalkanen et al. 2012b). However, large-scale comparisons to fuel reports of ship owners are constrained by the availability of vessel fuel reports. The capability of the model for estimating instantaneous power consumption has been evaluated to be moderately less accurate, with a mean prediction error of 15 % in a thorough case study (Jalkanen et al. 2012b). The evaluated emissions also agree fairly well with the results of several measurement campaigns presented in literature, for various engines, engine loads, and pollutants. A more detailed description of the model evaluation studies are presented by Jalkanen et al. (2009, 2012b).

Some of the most notable, recognized sources of uncertainty in the shipping emission modeling using the STEAM model have been described in Table 1. In regional-scale emission inventories, the most significant inaccuracies can be caused, e.g., by major temporal gaps in the received AIS data, the neglect of sea ice cover conditions (at present, the model does not include that), and the uncertainty concerning the ship fuel sulfur content. The evaluation of the chemical constituents of particulate matter can also be substantially uncertain. Clearly, the significance of inaccuracies vary depending on the spatial and temporal scale; for instance, the predictions of a journey of a single vessel may be significantly affected by stormy sea, but the accuracy of an annual regional inventory may not be substantially influenced by storms. The relative significance of these uncertainties also substantially varies in terms of the time period and geographical domain; e.g., the effects of the sea ice cover are more important in the northernmost parts of the Baltic Sea.

**Table 1:** Most notable sources of uncertainty, and their estimated significances in the evaluation of shipping emissions using the STEAM model. The relative contributions to the uncertainties of the predicted emissions have been categorized as minor, moderate, and major. These uncertainties correspond to those commonly occurring in annual average regional-scale evaluations; for specific ships or journeys, the relative significance of these uncertainties can be substantially different

Source of uncertainty	Characterization	Significance of uncertainty
Uncertainties of model input data	Major gaps in the geographical or temporal coverage of AIS data	Major. If there are major gaps in the relevant AIS data, there will be also major uncertainties in the predicted emissions
	Unavailability of AIS service due to infrequent technical failures	Minor. The STEAM model includes a treatment to interpolate over moderate signal gaps
	Poor AIS data quality	Minor. This concerns mainly satellite-based AIS data sets. The STEAM model can mitigate such effects by filtering out most of the erroneous information
	Incomplete or missing technical data for ships	Minor. The STEAM model will resort to using averages and rules-of-thumb to provide educated guesses for missing data. Data coverage can also be improved by combining various data sources
	Small vessel traffic not entirely accounted for	Minor. The contribution of small vessels to total emissions is limited, and these have been included in the model in an approximate manner
	Missing technical data for small vessels	Minor. This will cause some uncertainty only for the small vessel contribution, which is about 10 % of total CO_2_ emissions
Uncertainties of the prediction of power	Neglect of environmental effects (wind, waves, sea ice cover, currents)	From minor to major. These uncertainties can be significant for individual ships, but the impact on regional-scale predictions is likely to be small. Sea currents and sea ice cover can have a significant impact on ship emissions in specific regions
	The estimation of engine load	Minor. The STEAM model assumes identical main engines. If in reality engines do not have an equal power, inaccuracy in load balancing may occur
Uncertainties in evaluating the emission factors	Use of IMO Tier I NO_*x*_ curve for old vessels	From minor to moderate. This will underestimate NO_*x*_ emissions from old vessels, but it can be improved if more experimental data becomes available
	Insufficient experimental data on the chemical composition of particulate matter emissions	The formation of particulate matter emissions is a complex process. The STEAM model uses PM emission factors based on the most recent literature. However, the chemical composition is measured in different ways in various experimental setups
Fuel properties	Assumption of SECA/sulfur directive compliance	Moderate. Uncertainty in fuel sulfur content will have an impact on the predicted SO_*x*_ and PM emissions

### The AIS Data and the Technical Specifications of the Ships

The AIS position reports that were used for the study period (2006–2009) consist on the average of more than from 170 to 260 million messages each year (Table 2). There is an increasing trend in the number of received messages. Although the installation of AIS transmitters is voluntary for small vessels, their use in small vessels is increasing.

**Table 2:** The numbers of the archived AIS messages and active (operational) ships in the Baltic Sea in 2006–2009. The numbers of AIS messages in the table are lower level estimates. Active IMO-specified ship refers to a ship with an IMO number. The overall average temporal coverage of the AIS signals, and the percentages of IMO-specified and other active ships have also been presented

Years	Archived AIS messages (lower limits)	Temporal AIS coverage on the average (%)	Number of active ships	Number of active IMO-specified ships (%)	Number of active ships without the IMO number (%)
2006	>171 966 000	93.36	8160	6851 (84.0)	1309 (16.0)
2007	>210 345 000	97.90	9326	7355 (78.9)	1971 (21.1)
2008	>247 793 000	96.13	10 589	7311 (69.0)	3278 (31.0)
2009	>261 088 000	99.20	11 606	7422 (63.9)	4184 (36.1)

There were temporal gaps in the AIS data that varied from about 1 % (2009) to 7 % (2006) annually. However, their influence on both the location of the vessels and the total emissions has been taken into account, using linear interpolation for each gap, based on the existing data from the preceding and subsequent periods. The most significant gap in the data occurred in June 2006, when data during 2 weeks was missed, due to technical problems in signal reception of the Baltic Sea AIS network. The data during June 2006 has therefore a larger uncertainty than the corresponding data for the other months addressed. In addition to this major gap, there were AIS blackout periods of a few hours that occurred in some cases several times a week.

Non-active ships have been defined here as ships, which do not send more than one AIS-message in a month or do not consume any fuel according to the model (i.e., these are non-operational during the period considered). “Ships with IMO number” refer to the ships, for which it is mandatory both to use of AIS equipment and to registrate with the IMO, to obtain a unique registry number. Active ships with an IMO number represent the regular and registered marine traffic, whereas ships without an IMO number represent unregistered vessels.

Almost all IMO-specified ships encountered in the archived AIS data are included in the model’s internal database, together with their available technical specifications. Their annual fractions of all active ships varied from 64 % (2009) to 84 % (2006). For majority of ships without an IMO number, no reliable specification data has been available; these have therefore been assumed to be so-called small vessels. This designation of small vessels has been applied in cases, in which only vessel name and the MMSI code are available. In most of these cases, an international classification procedure is not required.

For the small vessels, average values representing the tugboats in the Baltic Sea have been used. However, this procedure is likely to slightly overestimate the contribution from small vessels, as tugboats commonly have larger engines than an average recreational craft. We have therefore cross-checked the national boat and radio license registers with the AIS data. This checking shows that there are very few large vessels (such as barges) among the vessels that have been assumed to be small.

The incomplete inclusion of small vessel traffic without AIS onboard probably tends to lead to a small underestimation of total emissions in this study. To avoid any bias in evaluating the annual trends of emissions, we have therefore reported separately (i) the emissions from all vessels, as well as (ii) the contribution from large vessels with an IMO number. We have also reported separately the temporal trends for small and large vessels.

**Fig. 1:**
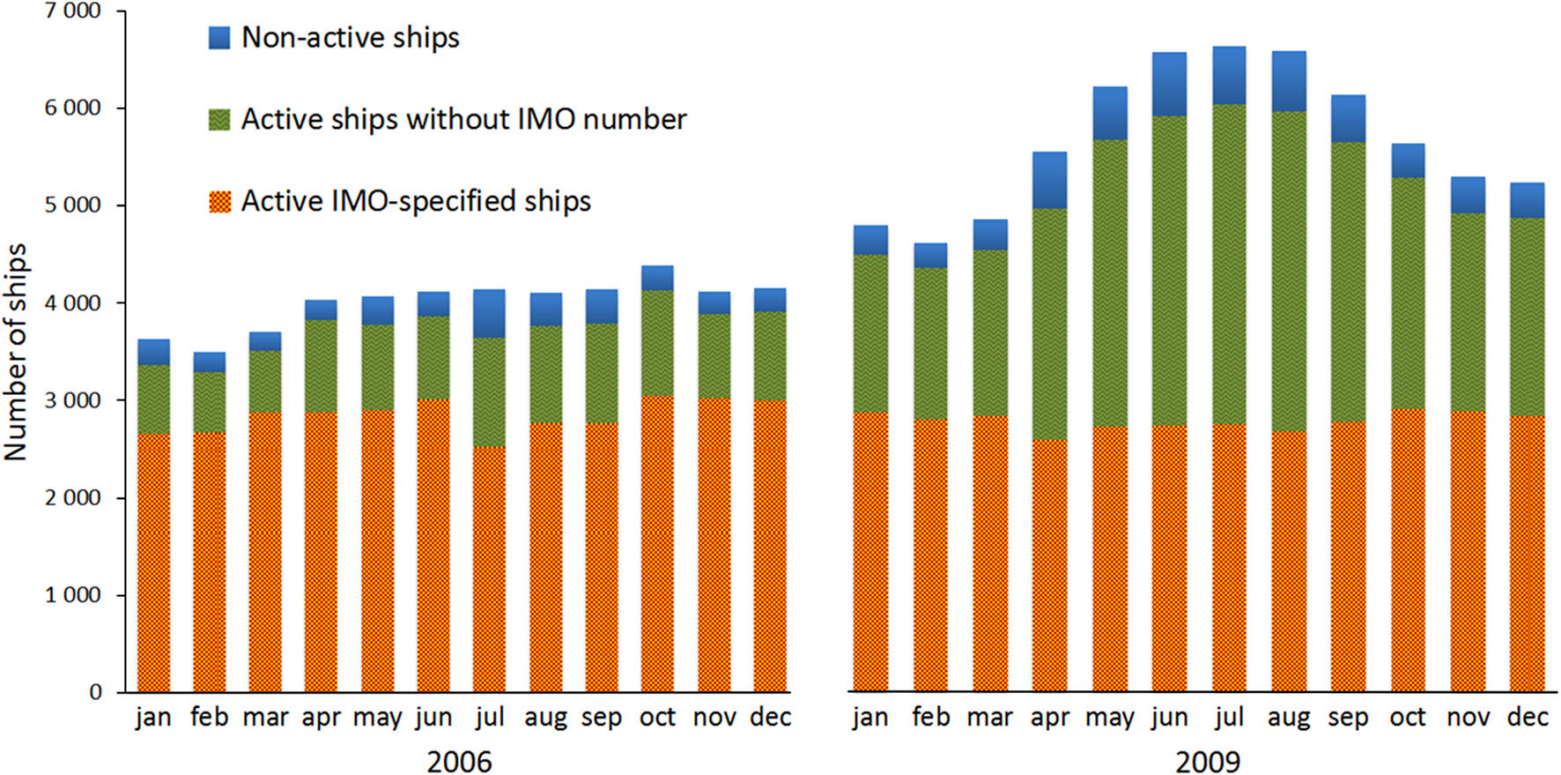
Seasonal variation of the numbers of the various categories of ships in the archived AIS data in 2006 and 2009

Both the total annual and the monthly numbers of ships are significantly higher in 2009, compared with those in 2006 (Fig. 1). However, the numbers of IMO-certified ships have remained almost the same. In 2009, 64 % of the ships represent the commercial and regular ship traffic. The regular and registered cargo ship traffic has no significant seasonal variation either in 2006 or 2009. However, in 2009, both the number of active ships without an IMO number and the number of non-active ships have a seasonal dependency; this is caused by the increased passenger and yacht traffic in summer in the Baltic Sea.

## Results and Discussion

### Overview of Emissions from Baltic Sea Shipping in 2006–2009

**Fig. 2:**
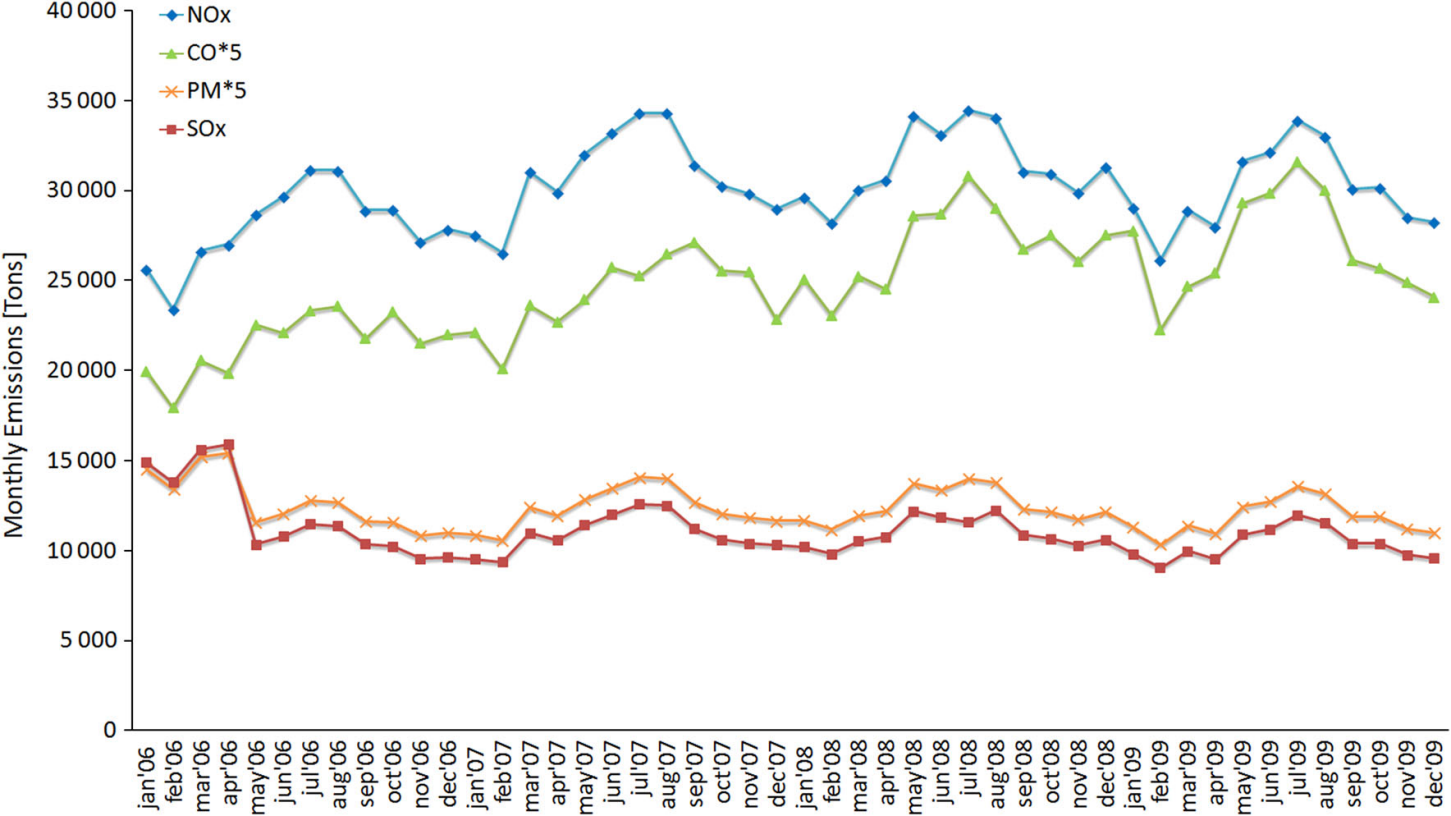
Predicted monthly emissions of NO_*x*_, CO, PM, and SO_*x*_ in 2006–2009. The PM and CO emissions have been multiplied by 5 for presentation purposes

The predicted annual emissions in 2006–2009 are listed in Table 3. The predicted monthly time series for NO_*x*_, SO_*x*_, PM, and CO emissions are presented in Fig. 2. In 2006, 15.6 × 10^9^ kg of CO_2_ was emitted by the marine shipping in the Baltic Sea according to Table 3; less than 6 % of these resulted from the ship traffic without certified IMO number.

**Table 3:** Predicted emissions in the Baltic Sea in 2006–2009, presented separately for all ships within this inventory, and for IMO-specified ships, for the selected pollutant gases and particulate matter. All annual emissions are presented in 10^6^ kg. Total PM_2.5_ emissions are assumed to be equal to the sum of OC, EC, ash, and SO_4_ together with its associated water. The category “All ships” (within this inventory) includes also small vessels without certified IMO number; detailed vessel specifications have not been available for most of this category of ships

	Emissions as predicted by STEAM (10^6^ kg)		2006	2007	2008	2009
All ships	Gaseous pollutants	CO_2_	15 600	15 900	16 600	15 900
		NO_*x*_	336	369	377	360
		SO_*x*_	144	132	132	124
		CO	51.6	58.1	64.5	64.3
All ships	PM_2.5_		29.1	27.6	25.5	23.3
	The chemical constituents of PM_2.5_	OC	5.7	6.3	6.5	6.2
		EC	2.2	2.4	2.5	2.4
		Ash	1.6	1.8	1.8	1.7
		SO_4_	20.9	19.1	19.2	18.0
IMO-specified	Gaseous pollutants	CO_2_	14 700	14 600	15 000	13 700
		NO_*x*_	321	345	345	318
		SO_*x*_	138	123	121	110
		CO	47.7	52.3	56.5	52.8
IMO-specified	PM_2.5_		29.1	27.6	25.5	23.3

The comparisons of the present results with those in other regional inventories of ship emissions have been reported in Jalkanen et al. (2012b). In general, the predicted STEAM emissions for NO_*x*_ differ by 8–14 % of the annual emissions reported by EMEP. Larger differences (up to 70 %, depending on the pollutant) can be found in predicted emissions of SO_*x*_, PM, and especially CO, due to different activity data sets and approaches used in the modeling. In the STEAM approach, international regulations (IMO, EU) for marine fuels have been taken into account. Predictions for CO are crucially dependent on the operation of marine engines. For instance, large diesel engines emit only moderately CO when operated using optimal engine loads, but the CO emissions may substantially increase, when engine load rapidly changes. This includes situations, in which vessel speed changes as a result of acceleration or active deceleration. However, in the construction of the EMEP ship emission inventories, vessel speed information was not available.

The contribution of the traffic in Baltic Sea to the global CO_2_ budget can be estimated to be 1.6 %, according to the second IMO greenhouse gas study, in which the total CO_2_ emissions were estimated as 1.046 × 10^12 ^kg in 2007 (Buhaug et al. 2009). Throughout the period considered, the predicted contribution from non-commercial traffic to total CO_2_ emissions has continually increased, which has been caused by the significant increase of small vessels and their activity (Table 2).

From 2006 to 2008, the total emissions of CO_2_, CO_2_, and NO_*x*_ were increasing, but decreased substantially in 2009, mainly caused by economic recession. The recession started in 2008 and continued throughout 2009, during which the CO_2_ emissions from commercial vessels decreased by 7 % (Table 3). Most of the Baltic Sea riparian states showed decreasing gross domestic product (GDP) rates since the second quarter of 2008, with only a couple of exceptions. In Denmark, the economic difficulties began already in the second quarter of 2006. In Russia, the recession started with a delay: the inflation adjusted annual GDP growth rate decreased 9 % in the second quarter of 2009 (Trading economics 2011).

Regular passenger traffic was least affected by the recession, whereas there was a significant decrease in all of the emissions from container ships and vehicle carriers. Indeed, the results show a decrease of 16 % in total cargo freight volume between 2006 and 2009. The cargo volume has been computed using ship type-specific fraction of deadweight, given by VTT (2011).

The increase in CO emissions in 2006–2008, and only a slight decrease from 2008 to 2009, is probably due to an increased number of other than IMO-specified ships; such smaller boats produce relatively larger amounts of CO. However, the PM_2.5_ emissions from all the ships within this inventory and those from only IMO-specified vessels were identical in all the considered years (within the uncertainty limits), as presented in Table 3.

According to Table 2, the number of small vessels has almost quadrupled during the study period. The contribution of small ships in terms of CO_2_ emissions, however, has increased from 7 % to only 14 %. This increased contribution to the emissions has not been large enough to compensate for the effect of decreased commercial traffic from large ships.

The annual emissions of SO_*x*_ and SO_4_ in relation to the reference year of 2006 have evolved differently than the previously discussed CO, NO_*x*_, and CO_2_ emissions, due to the SECA [SO_*x*_ emission control areas (SECA)] fuel sulfur requirements. On May 19, 2006, the maximum allowed sulfur content of fuel used in the SECA area was decreased from 2.7 to 1.5 %. The effect of this reduction is clearly visible in the temporal evolution of the SO_*x*_ emissions in Fig. 2.

There are clear seasonal variations in the emissions of all considered pollutants (Fig. 2). For example, excluding SO_*x*_ in 2006, the emissions of NO_*x*_, SO_*x*_, PM, and CO in July are 16–25 % larger than the corresponding values in January. The main reason for this is not the seasonal increase in the numbers of ships without an IMO number (Fig. 1), but the increase in fuel consumption of the relatively larger IMO-certified ships, for e.g., the Roll-on/Roll-off passenger ships (RoPax) are significantly more active in summer compared with winter. However, for the activities of most other ship types (except for RoPax and passenger ships), there is no substantial seasonal dependency.

### Geographical Emission Changes from 2006 to 2009

**Fig. 3:**
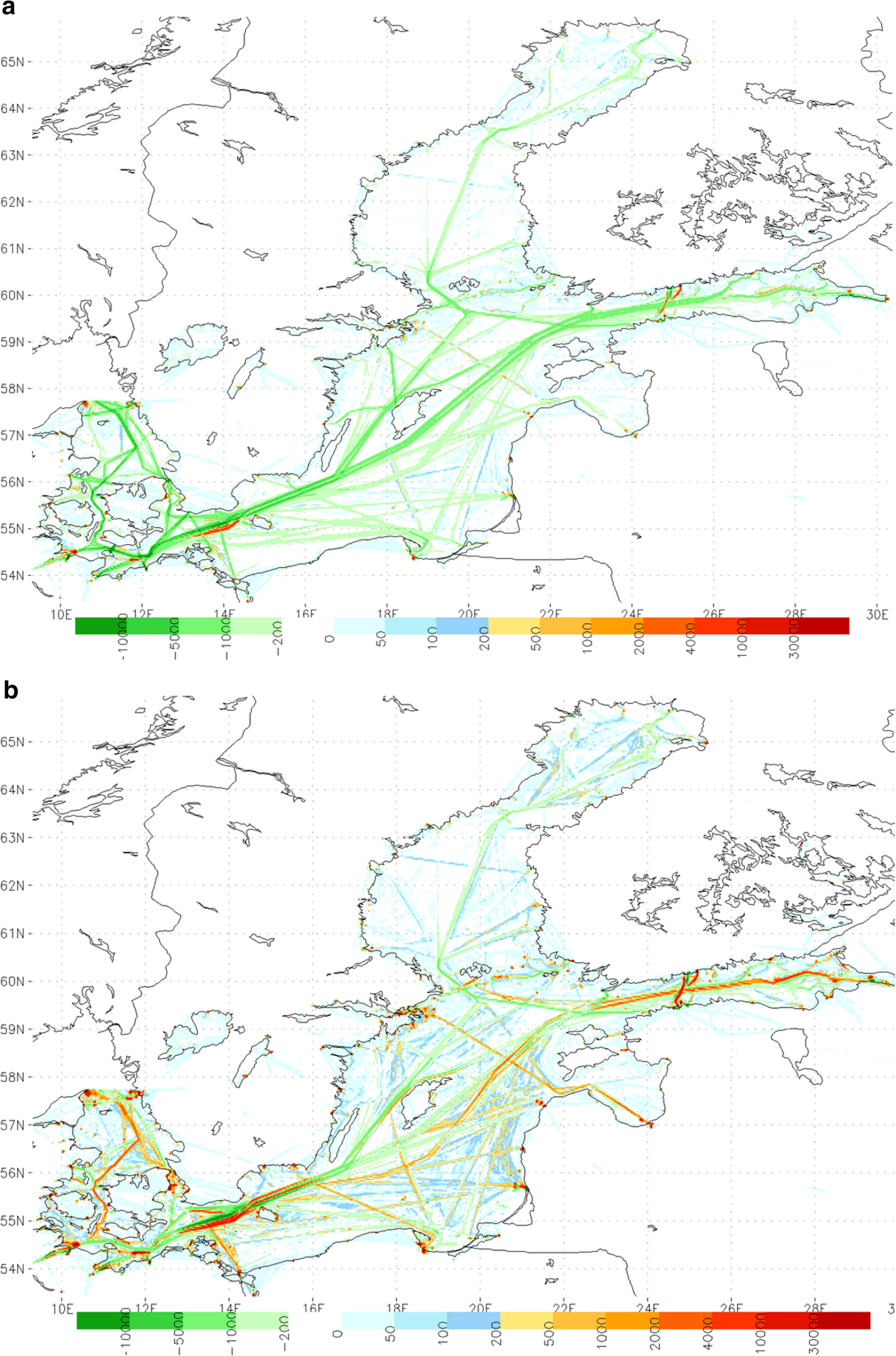
Predicted change of the spatial distribution of SO_*x*_ (**a**) and NO_*x*_ (**b**) emissions between the values during January–April in 2006 and 2009. *Green color* indicates the areas where emissions in 2006 exceeded the emissions of 2009, while the *other colors* indicate a temporal increase in emissions. The legend refers to total emissions in kilograms in an area of 0.03 × 0.03 degrees (approximately 6.5 km^2^)

Selected geographical distributions of the modeled emissions computed by the STEAM1 and STEAM2 models have been previously presented by Jalkanen et al. (2009, 2012b). The geographical implications of the reduction of the maximum sulfur content in May 2006 was investigated with a SO_*x*_ difference map; the aggregate SO_*x*_ emission distribution resulting from shipping in January to April of 2006 was subtracted with the respective emissions between January and April in 2009. The resulting comparison is presented in Fig. 3a.

It can be seen from Fig. 3a that relatively the largest SO_*x*_ emission reductions have occurred in the major ship travel routes. However, the SO_*x*_ emissions have increased within and in the vicinity of some harbors, most notably in those at Gdansk and Kiel. One probable explanation for this result is that the auxiliary fuel consumption (consisting of 0.5 % sulfur in mass according to modeling), which is focused in and near harbor areas, is unaffected by the imposed sulfur reducing legislation. However, the increase of SO_*x*_ emissions near the Kiel Canal and near Bornholm is mainly due to the increase of commercial ship traffic in these regions.

The emissions have also increased on some other densely trafficked routes, such as the one between Helsinki and Tallinn and in some routes in the Danish Straits, probably caused by increased traffic flows. The substantial increase of emissions 15 km east of Helsinki has been caused by the opening of a new major harbor (in the district of Vuosaari); this change has transferred most of cargo traffic from Helsinki city center to a less densely populated area. The increase of small vessel emissions is not necessarily focused on main shipping lanes, as small vessels are not usually limited by vessel draught and water depth.

Substantially more routes and domains with increased emissions can be found in the corresponding difference map for NO_*x*_ (Fig. 3b). The relative changes in the NO_*x*_ emissions can be expected to reflect fairly accurately the changes in overall fuel consumption. It is therefore also possible to indirectly identify geographical changes in overall shipping activities and travel routes based on the results in this figure.

The NO_*x*_ emissions in 2009 substantially exceed the emissions in 2006 at numerous major ship routes, including especially new and rapidly developing routes, such as many routes in the Danish Straits and their vicinity, and in the Gulf of Finland. In addition, there are substantially increased NO_*x*_ emissions at many harbors in Denmark and Germany, and in the Stockholm, Helsinki, and St. Petersburg areas. Again, these increases have partly been caused by the increase in small and unidentified ships that mostly operate near the coastline.

### Analysis of Emissions in Terms of the Flag State

**Fig. 4:**
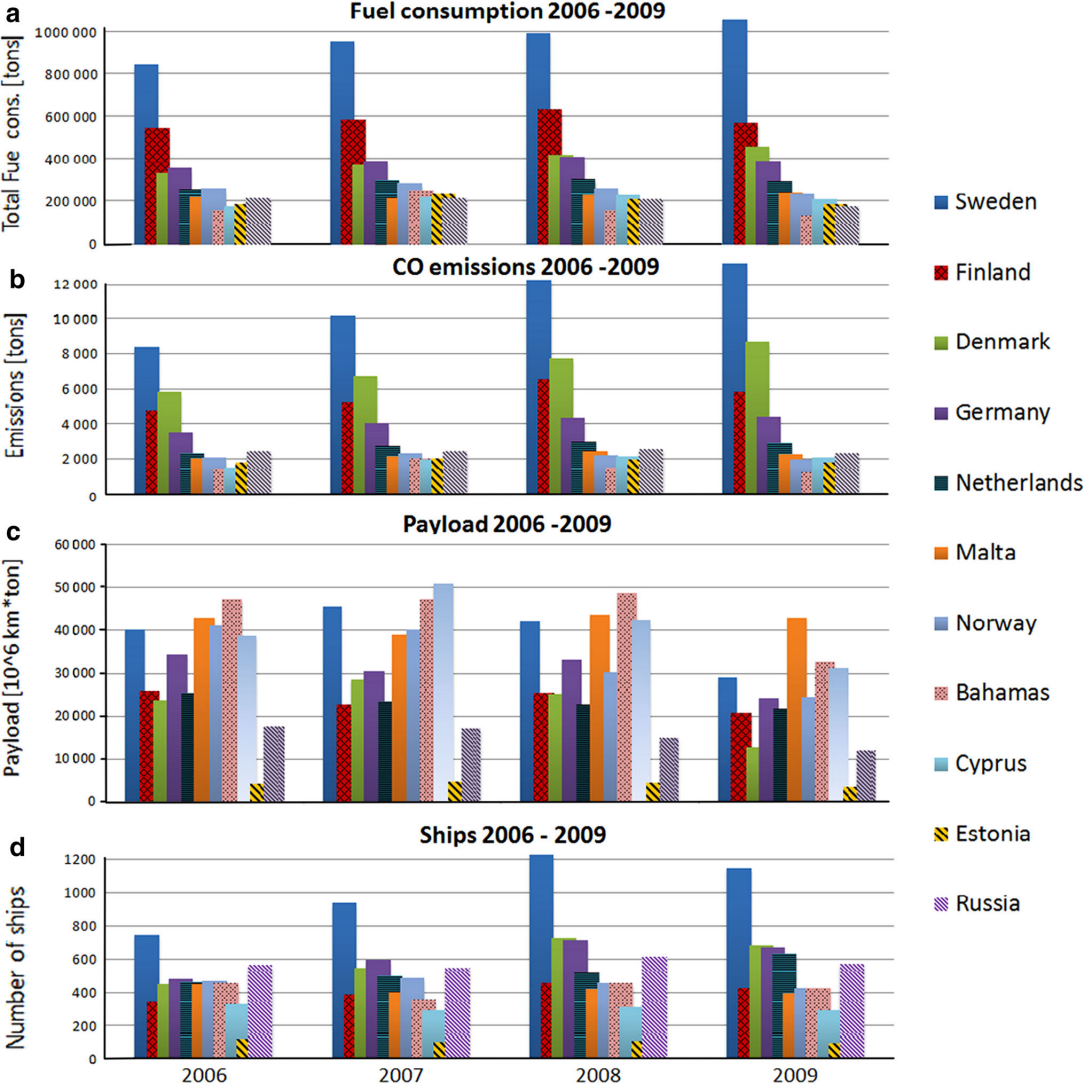
Predicted total fuel consumption (**a**), CO emissions (**b**), payload (**c**), and the number of ships (**d**) of 11 most contributing flag states in the Baltic Sea in 2006–2009

The AIS signals include a Maritime Mobile Service Identity (MMSI) code that contains information that specifies the flag state of the ship. We have selected 11 flag states that had the highest total fuel consumption in the Baltic Sea in 2006–2009, and presented their annual statistics of fuel consumption, CO emissions, payload, and the fleet size in Fig. 4a–d.

The total fuel consumption in all the considered years was largest for the Swedish and second largest for the Finnish fleet. The riparian states Denmark and Germany also have had major fleets. In addition to other riparian states (Estonia and Russia) and neighboring countries (the Netherlands and Norway), major fleets have also sailed in the Baltic Sea under the Malta, Bahamas, and Cyprus flags. The fuel consumption of the Russian fleet at the Baltic Sea was unexpectedly small.

The changes in fuel consumption of the riparian states are similar to their respective changes in GDP (such as the decreases from 2008 to 2009 for many countries), with a few exceptions. The Swedish and Danish fleets have steadily increased the fuel consumption throughout the whole period, despite the recession. The fuel consumption of the Russian fleet has steadily decreased. However, there is no decrease in the ship traffic near Russian harbors from 2006 to 2009 (Fig. 3b).

Computations have shown that the SO_*x*_, PM, and NO_*x*_ emissions of fleets correlate with the corresponding fuel consumption (data not shown here). The relative amounts of these emissions for the major flag states are similar to those of fuel consumption (Fig. 4a); these statistics have therefore not been presented here. However, the emissions of CO behave differently (Fig. 4b): e.g., the Danish vessels have been the second largest polluter of CO in the Baltic Sea. A relatively larger share of CO emissions indicates that a larger fraction of ships are small vessels. This is consistent with the substantial increase of the number of vessels (compared with the moderate increase of fuel consumption) in Denmark (Fig. 4a, d).

The ships sailing under the flag of Bahamas, Cyprus, and Malta were large, compared with those in other considered states. The average gross tonnage (GT) for Maltese and Bahamas ships were 11 000 and 14 000 tons, respectively; whereas the average weight for a Swedish ship was less than 2800 tons (values in 2009). The fleets of Bahamas, Cyprus, and Malta were also more cargo oriented (Fig. 4c).

The average ages of the fleets also significantly differ among the most contributing flag states. The Russian fleet was on the average the oldest in 2009, 25 years, and the fleets of Finland and Sweden were only slightly newer. The Netherlands had the youngest fleet, 11 years. The requirement of MARPOL Annex VI Tier I NO_*x*_ compliance will necessitate an upgrade of engines of 21 Finnish vessels with >5000 kW, built during 1990–1999. From these 13 are RoRo/RoPax vessels and 6 general cargo (GC) ships. In the Swedish fleet, 16 vessels will be affected, of which 11 are RoRo/RoPax vessels and 5 tankers. According to IMO MARPOL Annex VI, these vessels must be upgraded to meet Tier I NO_*x*_ requirements. However, the cylinder displacement requirement of 90 L was not taken into account, and the actual number of vessels in need of an engine upgrade may therefore be smaller than reported in this article.

### Analysis of Emissions in Terms of Ship Type and Size

According to the predictions, the marine traffic in the Baltic Sea is dominated by seven most contributing ship types. These seven types accounted for 93 % of both the total emissions of CO_2_ and fuel consumption in 2006–2009. The average properties of seven of these ship types have been summarized in Table 4.

**Table 4:** Selected characteristics of the most common ship types in the Baltic Sea marine traffic in 2009. *GT* Gross tonnage, *DWT* deadweight tonnage (the weight that the ship carries). Unit emission is the estimated amount of CO_2_ emissions per transferred payload and distance in km. For RoPax ships and containers, the unit emission is dependent on GT. *Avg* average

2009	RoPax	Tanker	General cargo	Container	RoRo	Bulk	Passenger
Average GT (ton)	16 560	27 380	4 680	20 770	15 010	25 800	18 440
Average DWT (ton)	3 290	47 140	6 390	24 060	9 030	44 600	2 110
Payload of DWT	0.42	0.5	0.4	0.4–0.65	0.24	0.5–0.6	–
CO_2_ unit emission (gton^−1 ^km^−1^)	127	8.51	30.6	26.0	67.7	7.32	–
Average age	19.7	8.6	15.8	8.5	15.5	13.9	30.4
Avg. main engine power (kW)	14 700	8 310	2 730	15 660	10 780	7 710	12 440
Avg. service speed (knots)	17.6	13	12.3	19.1	17	13.9	15
Common engine design	4-Stroke	2-Stroke	4-Stroke	Both	4-Stroke	2-Stroke	4-Stroke
Total ships in 2009 (change from 2006)	220 (−15)	1 785 (+316)	2 281 (−68)	347 (+106)	152 (−19)	984 (−100)	194 (+33)
Main purpose	Vehicle and passenger travel, cruising	Liquid cargo transfer	General cargo transfer	Container cargo transfer	Vehicle transfer	Bulk cargo transfer	Cruising and passenger travel

RoRo and RoPax ships, besides having several hundred passengers onboard, also transfer cargo on wheels, such as trucks and cars, whereas passenger ships are more leisure oriented and can accommodate up to several thousand passengers. The payload of these vessels is commonly small, compared with the cargo ships. The large Passenger ships travel in the Baltic Sea 10 months per year. In contrast, unspecified ships and bulk carriers travel infrequently, and these ships are used on the average only during 3 months.

**Fig. 5:**
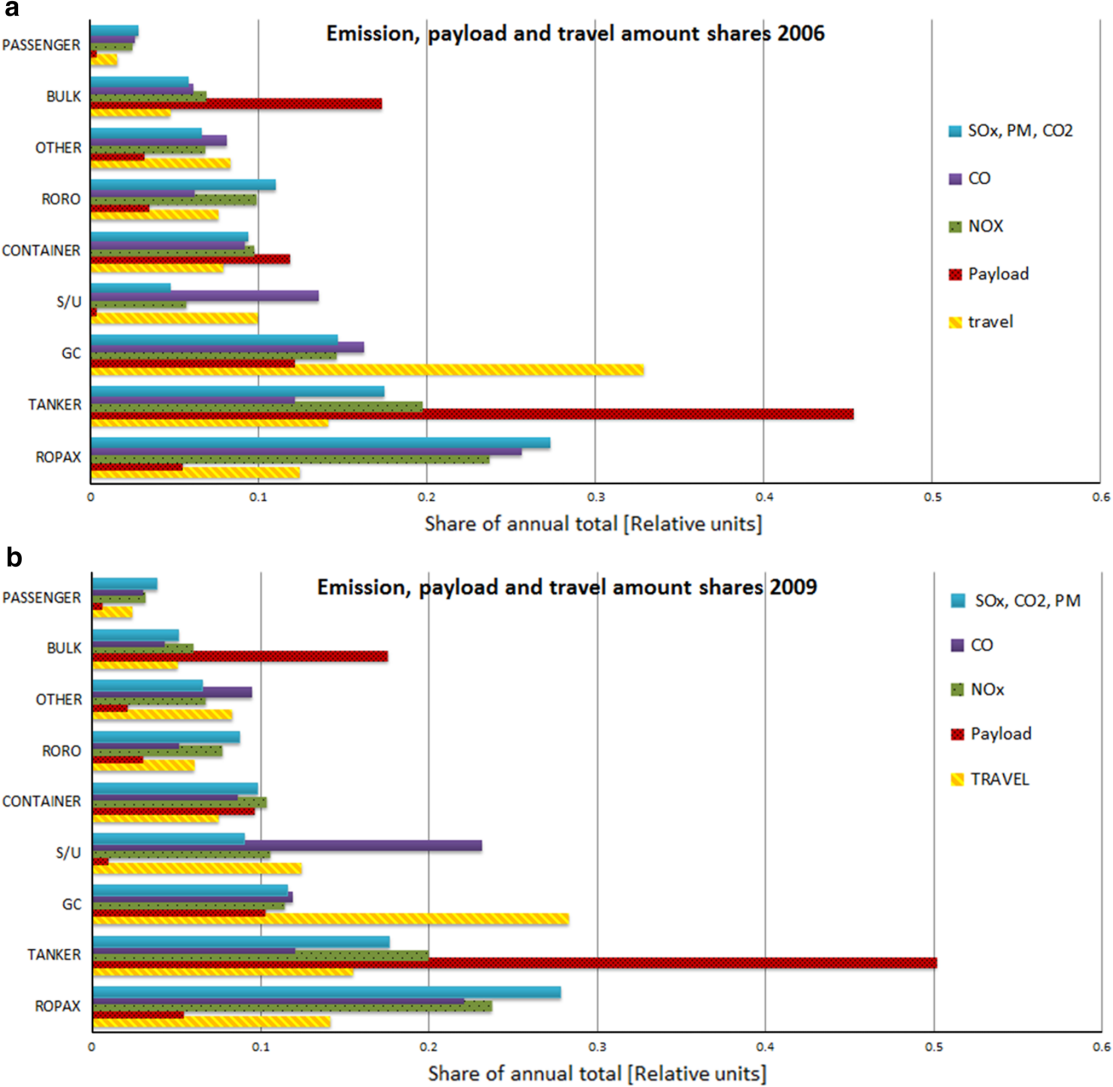
Fractions of emissions (for three types of pollutants), payload, and travel in the Baltic Sea in 2006 (**a**) and in 2009 (**b**) for the major ship types. The fractions of emissions of SO_*x*_, CO_2_, and PM were according to predictions approximately equal (differed at most ±1 % of the presented values), and have therefore been presented as one column only. S/U refers to small tugs and unspecified ships

These emission shares in 2006 and 2009 for each ship type are presented in Fig. 5a, b. The types of RoPax, tankers, and GC have had the highest emissions of the presented pollutants during all the years of this study (the results for 2007 and 2008 not presented here).

The heavy cargo ship classes are not responsible for most of the emissions. Bulk ships, which are the heaviest vessels sailing in the Baltic Sea, contributed of the order of 5 % of the total emissions, whereas the RoPax ships contributed approximately one-fourth.

**Fig. 6:**
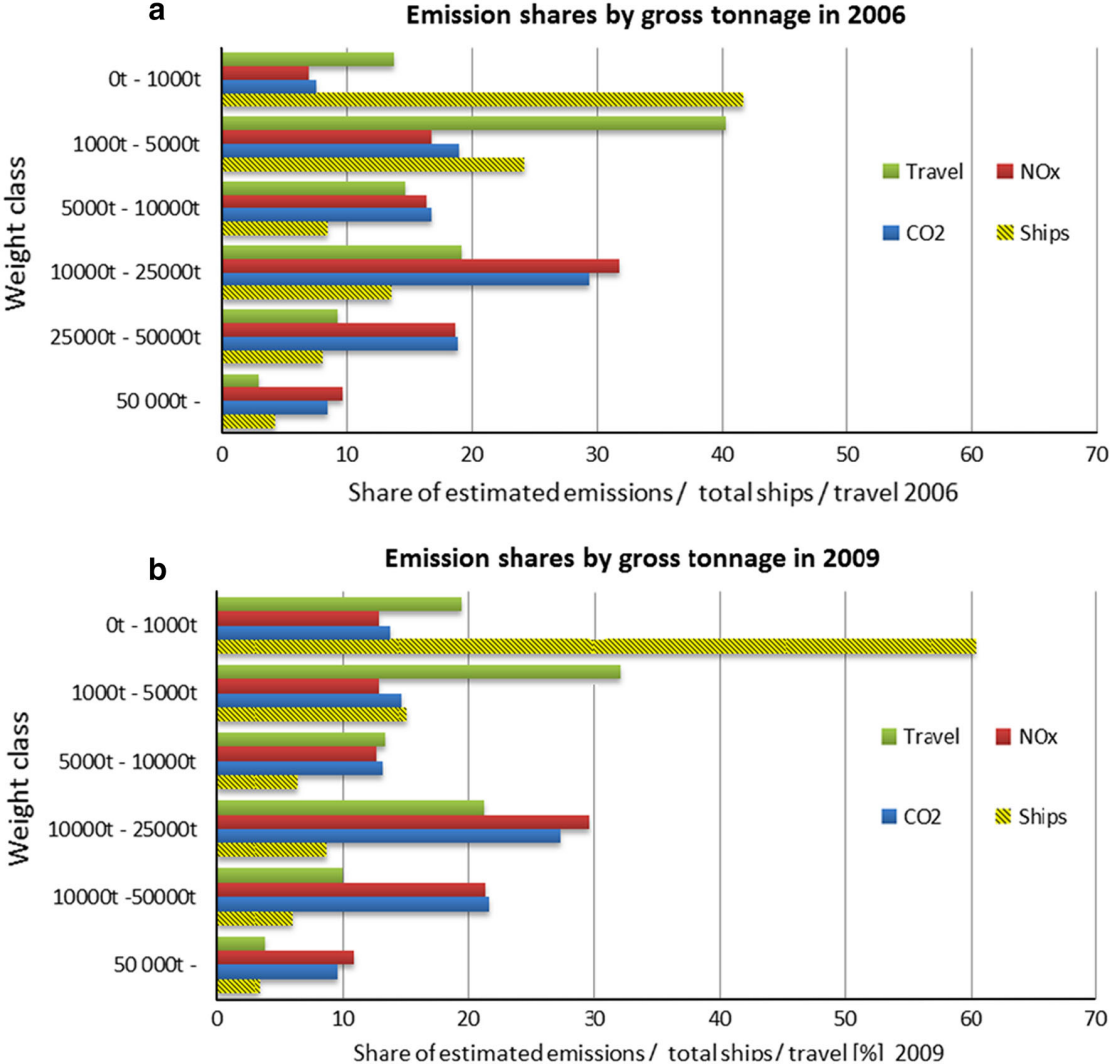
Relative emission, ship number, and travel distance distribution among ship weight classes in 2006 and 2009. Unidentified and presumably small vessels without IMO identification have been associated with 500 gross tons

To study the contribution on total emissions of the heaviest ships sailing in the Baltic Sea, the estimated emission shares were also allocated between different weight classes in 2006 and 2009 (Fig. 6). The results were that ships weighting more than 25 000 tons were responsible for approximately 27 and 33 % of the total PM, SO_*x*_, CO_2_ emissions in 2006 and 2009, respectively. The role of heaviest weight classes in the emissions has increased throughout the study period. Furthermore, despite the significant increase in small vessels and their activity, the heaviest weight classes presented in Fig. 6 have, in fact, increased their relative contribution between 2006 and 2009 at the expense of medium sized vessels.

In 2009, the majority of NO_*x*_ emissions (61.7 %) were produced by 2971 ships with GT more than 10 000 tons. Most of these are bulk carriers and tankers, which prefer 2-stroke engines, as these ships are heavy and require large power outputs. They are designed to run with relatively small revolutions per minute (RPM) and they produce relatively more NO_*x*_ emissions per produced engine power output contributing to the large share of NO_*x*_ from bulk carriers and tankers.

The relative usage of main engine fuel and auxiliary fuel is substantially different for the weight classes. For example, in 2009, the largest weight class (GT > 50 000 tons) is responsible for using 12.5 % of main engine fuel, but only 4.9 % of auxiliary fuel. Besides the weight of the ship, also the velocity is a crucial factor for the fuel consumption. The latter is one major reason for the fact that the RoPax ships (that have a highest average service speed of 18 knots) have the largest share of total emissions. On the other hand, bulk ships and tankers relative low speeds (lower than 14 knots) and large cargo capacity, which makes them the most economical classes in terms of the unit emissions per transferred payload (Table 4).

## Conclusions

This article has presented the most comprehensive review up to date on the emissions of CO_2_, NO_*x*_, SO_*x*_, CO, and PM_2.5_ originated from Baltic Sea shipping from 2006 to 2009. Numerical results have been computed using the Ship Traffic Emissions Assessment Model (STEAM2) that has been presented and evaluated in detail in previous studies (Jalkanen et al. 2009, 2012a,b, ). The AIS position reports that were archived and used for the study period consist on the average of more than 220 million messages each year. Another major technical database used in this study contains technical specifications for more than 45 000 ships from various sources of information, such as the data from IHS Fairplay (IHS Fairplay 2012). We expect that of these results will be useful both for the subsequent evaluation of the health effects caused by marine traffic and for the consideration of emission reductions by the IMO and other organizations.

Both the total annual and the monthly numbers of ships were significantly higher in 2009, compared with those in 2006. This has been mainly caused by the increased passenger and yacht traffic in the Baltic Sea, while the numbers of IMO-certified ships have remained almost the same. The economic recession, which started in 2008 and continued throughout 2009 in most of the Baltic Sea riparian states, had a clear impact on shipping emissions: despite the modestly growing trend in total NO_*x*_, CO_2_ and CO emissions between 2006 and 2008, total emissions in 2009 were ultimately comparable to the respective emissions in 2006. However, a detailed analysis revealed that the CO_2_ emissions from regular marine traffic had been reduced 7 %, while their aggregate cargo volume at the Baltic Sea was reduced by 16 %.

The annual emissions of SO_*x*_, SO_4_, and PM_2.5_ have evolved differently than those of CO, NO_*x*_, and CO_2_, due to the SECA fuel sulfur requirements. On May 19, 2006, the maximum allowed sulfur content of fuel used in the SECA area was decreased from 2.7 to 1.5 %. The effect of this reduction is clearly visible in the temporal evolution of the SO_*x*_ and PM emissions.

There are clear seasonal variations in the emissions of all considered pollutants. In general, the emissions of NO_*x*_, SO_*x*_, PM, and CO in July are 16–25 % larger than the corresponding values in January, but SO_*x*_ emissions of 2006 are an exception to this because of the fuel sulfur content change. The main reasons for these seasonal variations are the increased traffic and fuel consumption of the IMO-certified ships in summer.

Results have been presented on the differential geographic distribution of shipping emissions before (Jan–April 2006) and after (Jan–April 2009) the SECA regulations. Although the SO_*x*_ emissions have been reduced in the vast majority of routes in early 2009, compared with the early 2006, these have increased in the vicinity of some harbors, such as, e.g., those at Gdansk and Kiel. This is probably mainly caused by the auxiliary fuel consumption (consisting of 0.5 % sulfur in mass), which is focused in and near harbor areas, as it has been unaffected by the sulfur reducing legislation. The emissions have also increased on some densely trafficked routes, such as the one between Helsinki and Tallinn and in some routes in the Danish Straits, probably caused by increased traffic flows.

The NO_*x*_ emissions in 2009 substantially exceed the emissions in 2006 at numerous major ship routes, including especially new and rapidly developing routes, such as many routes in the Danish Straits and their vicinity, and in the Gulf of Finland. There are also substantially increased NO_*x*_ emissions at many harbors, and in the Stockholm, Helsinki, and St. Petersburg areas. The increases near major cities have partly been caused by the increase in relatively smaller ships that mostly operate near the coastline.

Numerical results were also presented on the emissions allocated in terms of flag state, ship type, and weight. The total fuel consumption in all the considered years was largest for the Swedish, Finnish, Danish, and German fleets. In addition to other riparian states (Estonia and Russia) and near-by countries (the Netherlands and Norway), major fleets have also sailed in the Baltic Sea under the Bahamas, Cyprus, and Malta flags. The ships sailing under the flag of Bahamas, Cyprus, and Malta were relatively larger and more cargo oriented, compared with those in other considered states.

The marine traffic in the Baltic Sea is dominated by the seven most contributing ship types. These seven types accounted for 93 % of both the total emissions of CO_2_ and the fuel consumption in 2006–2009. The types of RoPax, tankers, and GC have had the highest emissions of the presented pollutants during all the years of this study.

Whereas the emission levels of CO, NO_*x*_, and CO_2_ from all ships have increased during the considered period, the emissions of SO_*x*_ and PM have decreased by 14 and 7 %, mostly due to the SECA regulations of the IMO in May of 2006. However, the actual effect of the regulation is more significant, as the higher sulfur limit of 2.7 % was not applied throughout the whole base line calendar year of 2006.

The fuel sulfur reduction affected also particulate matter emissions, which decreased 7 % during the study period, as part of the gaseous sulfur oxidizes to particulate sulfate. After the time period considered in this study, a further reduction from 1.5 to 1.0 % of the maximum allowed sulfur content of the fuel was implemented on July 1, 2010. The detrimental health effects caused by shipping exhausts are closely connected to the emissions of PM. Further reduction of the emissions of SO_*x*_ and PM from shipping in the Baltic Sea is expected, caused by more stringent SECA regulations and the European sulphur directive 2005/33/EC. A further decrease of the fuel sulfur content to 0.1 % has been planned to be implemented on Jan 1, 2015.
